# Aberrant Lymphatic Endothelial Progenitors in Lymphatic Malformation Development

**DOI:** 10.1371/journal.pone.0117352

**Published:** 2015-02-26

**Authors:** June K. Wu, Christopher Kitajewski, Maia Reiley, Connie H. Keung, Julie Monteagudo, John P. Andrews, Peter Liou, Arul Thirumoorthi, Alvin Wong, Jessica J. Kandel, Carrie J. Shawber

**Affiliations:** 1 Department of Surgery, College of Physicians & Surgeons, Columbia University, New York, New York, United States of America; 2 Department of Ob/Gyn, College of Physicians & Surgeons, Columbia University, New York, New York, United States of America; 3 Department of Surgery, the University of Chicago Medicine, Chicago, Illinois, United States of America; Center for Molecular Biotechnology, ITALY

## Abstract

Lymphatic malformations (LMs) are vascular anomalies thought to arise from dysregulated lymphangiogenesis. These lesions impose a significant burden of disease on affected individuals. LM pathobiology is poorly understood, hindering the development of effective treatments. In the present studies, immunostaining of LM tissues revealed that endothelial cells lining aberrant lymphatic vessels and cells in the surrounding stroma expressed the stem cell marker, CD133, and the lymphatic endothelial protein, podoplanin. Isolated patient-derived CD133+ LM cells expressed stem cell genes (NANOG, Oct4), circulating endothelial cell precursor proteins (CD90, CD146, c-Kit, VEGFR-2), and lymphatic endothelial proteins (podoplanin, VEGFR-3). Consistent with a progenitor cell identity, CD133+ LM cells were multipotent and could be differentiated into fat, bone, smooth muscle, and lymphatic endothelial cells *in vitro*. CD133+ cells were compared to CD133− cells isolated from LM fluids. CD133− LM cells had lower expression of stem cell genes, but expressed circulating endothelial precursor proteins and high levels of lymphatic endothelial proteins, VE-cadherin, CD31, podoplanin, VEGFR-3 and Prox1. CD133− LM cells were not multipotent, consistent with a differentiated lymphatic endothelial cell phenotype. In a mouse xenograft model, CD133+ LM cells differentiated into lymphatic endothelial cells that formed irregularly dilated lymphatic channels, phenocopying human LMs. *In vivo*, CD133+ LM cells acquired expression of differentiated lymphatic endothelial cell proteins, podoplanin, LYVE1, Prox1, and VEGFR-3, comparable to expression found in LM patient tissues. Taken together, these data identify a novel LM progenitor cell population that differentiates to form the abnormal lymphatic structures characteristic of these lesions, recapitulating the human LM phenotype. This LM progenitor cell population may contribute to the clinically refractory behavior of LMs.

## Introduction

Vascular anomalies are a heterogeneous group of lesions with arterial, venous, or lymphatic components that develop *in utero* or shortly after birth. These lesions are classified into malformations and tumors, based on histological classification, endothelial cell morphology, and clinical behavior [1–3]. Vascular malformations are further classified based on the cellular subtype of the malformation, with lymphatic malformations (LMs) consisting of abnormal lymphatic vasculature. LMs are subdivided on the basis of morphology, and include macrocystic (lumen >1cm), microcystic (lumen <1cm), mixed macrocystic and microcystic (mixed), and diffuse LMs (referred to as generalized lymphatic anomalies, GLA) [2–4].

The lymphatic vasculature functions in maintenance of interstitial fluid balance, mounting immune responses, and uptake of lipids and lipid-soluble nutrients from the intestines. Consequently, individuals with LMs are subject to significant morbidities resulting from disruption of these essential functions, including lymphedema, lymphatic fluid pooling (chylous ascites and chylothorax), and intralesional bleeding. Mass effects of large LMs can impair vital functions, such as cervicofacial lesions that cause airway obstruction or impingement on the eye. Superinfection of tissues in which lymphatic flow is impaired can lead to overwhelming sepsis. Despite this significant burden of disease, the pathobiology of these lesions is poorly understood.

LMs are frequently refractory to treatment. Often LMs cannot be removed in their entirety by surgery, because they infiltrate normal tissues. Ablation by sclerotherapy is limited to macrocystic areas. Thus, recurrence is common: 22–26% after surgery and 57% after sclerotherapy [5, 6]. Lymphatic anomalies have been associated with mutations in VEGFR-3 (Milroy’s disease), RASA1 (Capillary lymphatic-arteriovenous malformations, CLAVM), Foxc2 (Lymphedema-Distichiasis syndrome) and PTPN11 (Noonan’s syndrome) [7–10]. However, the molecular causes of the majority of LMs remain unknown, hindering development of biologically-targeted therapies.

Here, we identify a previously undescribed lymphatic malformation progenitor cell (LMPC) population in LMs that line the aberrant lymphatic vessels and reside in adjacent parenchyma in patient tissues. In LM tissues, LMPCs co-expressed the stem cell marker, CD133, and the lymphatic endothelial cell (LEC) marker, podoplanin. CD133^+^ cells isolated from LM patient tissues and fluid aspirates were multipotent, and expressed markers of stem cells, circulating endothelial precursor cells, and LECs. We compared CD133^+^ LM cells to CD133^−^ LM cells isolated from LM fluids. Relative to CD133^+^ LM cells, CD133^−^ LM cells had significantly lower expression of stem cell markers, maintained expression of circulating endothelial precursor markers, and expressed increased levels of differentiated LEC markers. Unlike CD133^+^ LM cells, CD133^−^ LM cells were not multipotent, suggesting that CD133^−^ LM cells represent differentiated lymphatic malformation endothelial cells (LMECs). We demonstrate that LMPCs recapitulate the LM phenotype in a mouse model. When xenografted in mice, CD133^+^ LM cells differentiated into LECs that formed aberrant lymphatic vessels, morphologically and histologically similar to those observed in LM patient tissues. Taken together, these data suggest a progenitor cell origin for human LMs.

## Materials and Methods

### Clinical Samples

Resected tissues and aspirated fluids were acquired from pediatric LM patients (infants to adolescents). For histologic and molecular characterization, tissues were fixed in 4% paraformaldehyde, incubated in 20% sucrose/PBS, and frozen in OCT or fixed in formalin and paraffin-embedded. Cells were isolated immediately from resected tissues and aspirated fluids. Description of LM specimens and methodologies performed are presented in Table S1 in S1 File.

### Cell Culture

LM cells were isolated from tissues or centrifuged fluids using the anti-CD133 bead selection system (Miltenyi Biotec) as described [11, 12]. Cells were maintained in EGM-2 media (Lonza) supplemented with 18% FBS on fibronectin-coated plates. Fluorescence-activated cell sorting (FACS) of CD133^+^ live cells was performed to generate CD34-positive or negative populations, and podoplanin-positive or negative populations. Hemangioma stem cells (HemSC) and human dermal lymphatic endothelial cells (HdLECs) were isolated and maintained as described [11–13]. Human bone marrow-derived mesenchymal stem cells (MSCs) were purchased and maintained as described by the manufacturer (Lonza).

For differentiation assays, cells were plated at 50% confluency on fibronectin-coated wells and grown in differentiation media for 14 days. Adipocyte differentiation media consisted of 1μg/ml insulin, 1μM dexamethasone, 0.5mM isobutylmethylxanthine, and 60μM indomethacin in 10%FBS/DMEM (low glucose) containing 1x penicillin-streptomycin. Adipogenesis was confirmed by Oil Red O staining. Osteoblast differentiation media consisted of 60μM ascorbic acid-2-phosphate, 10mM β̃glycerophosphate, and 1μM dexamethasone in 10%FBS/DMEM containing 1x penicillin-streptomycin. Osteogenesis was determined by alkaline phosphatase reaction. Mural cell differentiation media consisted of 10ng/ml TGFβ1 in 10% FBS/DMEM containing 1x penicillin-streptomycin. Mural cell differentiation was determined by α̃smooth muscle actin staining (Table S2 in S1 File). LEC differentiation media consisted of 10ng/ml VEGF-B, 10ng/ml VEGF-C, 10μl/ml insulin-transferrin-selenium, 10μl/50ml linoleic acid-albumin, 1nM dexamathasone, 60μM ascorbic acid-2-phosphate in EBM-2 (Lonza) containing 1x penicillin-streptomycin [14]. LEC differentiation was determined by CD31, VE-cadherin, LYVE1, and podoplanin immunostaining and by qRT-PCR.

### Immunohistochemistry (IHC)

Frozen and paraffin-embedded sections were immunostained as described [15, 16]. Cultured cells were fixed in 4% paraformaldehyde and stained as described [11]. Primary antibodies included α-smooth muscle actin (αSMA), CD133, CD31, GFP, LYVE1, NG2, Prox1, VE-cadherin, and VEGFR-3 (described in Table S2 in S1 File) and were detected with secondary Alexa Fluor antibodies (Invitrogen). Mouse-on-mouse kit (Vector Labs) was used with mouse monoclonal antibodies in murine LM implants. Images were captured with a Nikon Eclipse E800 microscope and Nikon DXM 1200 digital camera, using ImagePro Plus v.4.01 software, or a Zeiss Axioskop2 Plus and Zeiss AxioCam MRc camera with Zeiss Zen software.

### Quantitative RT-PCR (qPCR)

RNA was isolated (RNeasy Mini Kit, Qiagen) and cDNA synthesized (SuperScript First-Strand Synthesis System, Invitrogen) [17]. qPCR with Sybr Green Master Mix (ABI) was performed in triplicates using a CFX96 PCR Cycler (Bio-rad). Gene-specific PCR products cloned into pDrive (Stratagene) served as standards and reactions were normalized to β-actin. Primers are listed in Table S3 in S1 File.

### Fluorescence-activated cell sorting (FACS)

FACS antibodies included CD11b, CD146, CD31, CD34, CD90, podoplanin, VE-cadherin, VEGFR-2, and VEGFR-3 (described in Table S2 in S1 File). After antibody incubation, cells were disaggregated through a cell strainer to generate a single cell suspension. FACS was performed using FACSCalibur and CellQuestPro acquisition software (BD Biosciences) and the BD FACSCalibur flow cytometer and Cellquest Pro software. For live cell sorting, 2.5 x 10^7^ CD133^+^ LM cells were incubated with an antibody against CD34 or podoplanin (described in Table S2 in S1 File), single cell suspensions generated, and single cells sorted using BD FACSAria Cell Sorter (n = 3 CD133^+^ LM populations).

### Mice

Immunocompromised C57Bl *Rag2* ^*-/-*^ *;IL-2*γ*C* ^*-/-*^ *;CBP* ^*eGFP/+*^ were generated by crossing *CBP* ^*eGFP/+*^ mice (Jackson Labs) with *Rag2* ^*-/-*^ *;IL-2*γ*C* ^*-/-*^ mice (Taconic). LMPCs (1.5x10^6^ or 3x10^6^ cells) were resuspended in 200μl or 400μL Matrigel Matrix Phenol Red-Free (BD) and injected into the flanks of C57Bl *Rag2* ^*-/-*^ *;IL-2*γ*C* ^*-/-*^ *;CBP* ^*eGFP/+*^ mice or Nude (Nu/J) mice (n = 6 for each LMPC population). Control mice received MSCs, HemSCs, HdLECs, or Matrigel alone (n = 5–8). Mice were sacrificed at 3 and 5 weeks, and the implants harvested and paraffin-embedded.

### Ethics Statement

For human studies, de-identified tissues and aspirated fluid were collected from discarded surgical specimens. As protected health information (PHI) was neither stored nor disclosed to the researchers, this study was deemed exempt by Columbia University IRB (AAAA7338). Therefore, there was no verbal or written consent required. Mouse studies were approved by Columbia University IACUC (AC-AAAC1609 and AC-AAAG5852).

## Results

### LM tissues contain CD133^+^ stromal and lymphatic endothelial cells

**Fig 1:**
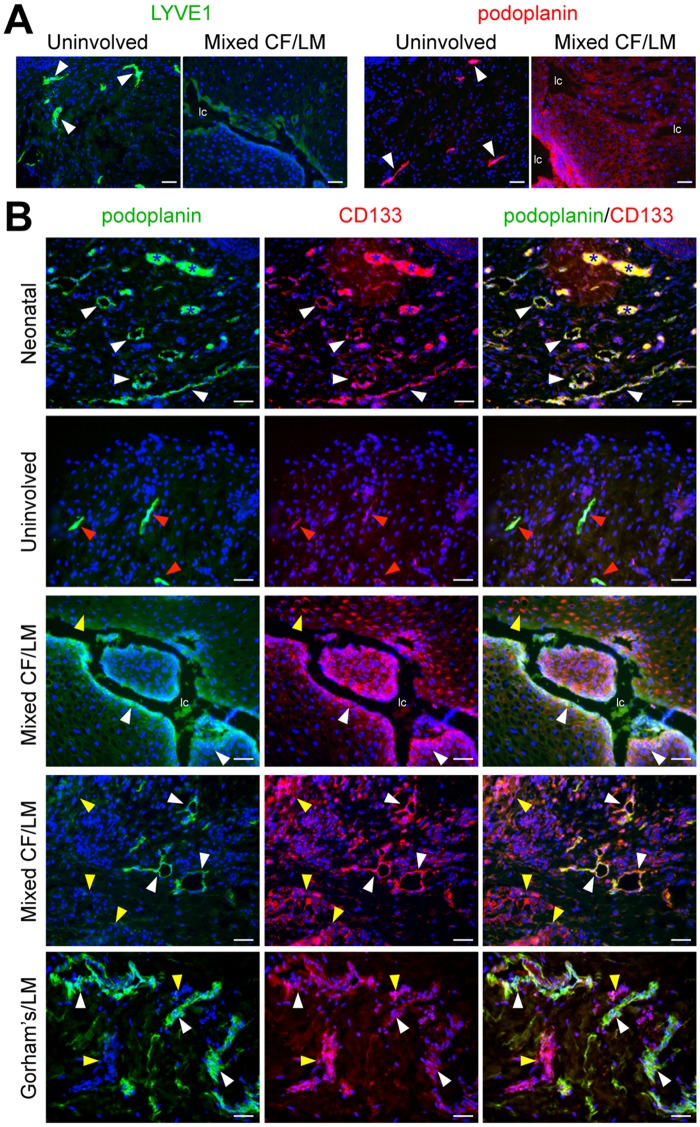
Identification of CD133^+^ cells in LMs of different subtypes and anatomical locations. (A) LYVE1 and podoplanin staining of cervicofacial mixed LM tissue and patient-matched uninvolved tissue. White arrowheads mark normal lymphatics. (B) Podoplanin and CD133 staining of neonatal foreskin (postnatal day 1), uninvolved tissue, mixed cervicofacial (Mixed CF) LM tissues (2x), and Gorham’s dermal tissue. White arrowheads mark CD133^+^/podoplanin^+^ lymphatic endothelium. Red arrowheads mark CD133^low^/podoplanin^+^ lymphatic endothelium. Yellow arrowheads mark CD133^+^/podoplanin^+^ stromal cells. Blue asterisks mark blood vessels with autofluorescing red blood cells. Scale bars: 50μm. lymphatic channel (lc)

Involved and uninvolved tissues resected from a patient with a mixed cervicofacial LM were stained for LEC markers, podoplanin and LYVE1. In uninvolved tissues, morphologically normal lymphatic vessels stained strongly for LYVE1 and podoplanin (Fig. 1A). In LM tissues, cells lining the irregularly dilated or ectatic lymphatic channels stained weakly for LYVE1 and strongly for podoplanin (Fig. 1A, Fig. IA in S2 File). In the LM tissue parenchyma, stromal cells stained weakly for podoplanin. These podoplanin^+^ stromal cells had a mesenchymal morphology, potentially consistent with that of an LM progenitor cell.

Circulating endothelial precursor cells have been shown to express CD133 [18, 19]. We determined CD133 and podoplanin expression in LM tissues from 7 patients, as well as in control neonatal foreskin (postnatal day 1), and 3 patient-matched uninvolved tissues. In neonatal foreskin, lymphatic vessels expressed CD133 consistent with the lymphatic vessel immaturity and active remodeling proposed to occur shortly after birth (Fig. 1B, Figs. IA, IB in S2 File)[20]. In contrast, CD133 expression was very low in the mature lymphatic vessels in the uninvolved tissue (Fig. 1B). The ectatic lymphatic endothelium and the cells lining the lymphatic channels in LMs expressed both CD133 and podoplanin (Fig. 1B). In the parenchyma, two different sub-populations of CD133^+^ cells were observed, CD133^+^/podoplanin^low^ and CD133^+^/podoplanin^−^ cells. Parenchymal CD133^+^ cells were detected in all LM tissues evaluated (mixed cervicofacial LM (n = 2), macrocystic subcutaneous LMs (under the skin; n = 2), macrocystic mesenteric LM (n = 1), Gorham’s LM (n = 1), generalized lymphatic anomalies (GLA; n = 1) (Fig. 1B, data not shown). CD133 expression in the lymphatic endothelium in LMs was similar to the immature neonatal lymphatic vasculature, suggesting arrested or defective development of LM lymphatics. In contrast, CD133 expression was only weakly expressed in normal LECs, consistent with their mature status. CD133^+^/podoplanin^+^ cells were observed in several different subtypes of LMs from various anatomic locations. Despite this anatomic and subtype heterogeneity, our data indicates that these CD133^+^/podoplanin^+^ LM cells are common to multiple lymphatic anomalies.

### CD133^+^ LM cells express markers of stem cells and endothelial cell precursors

**Fig 2:**
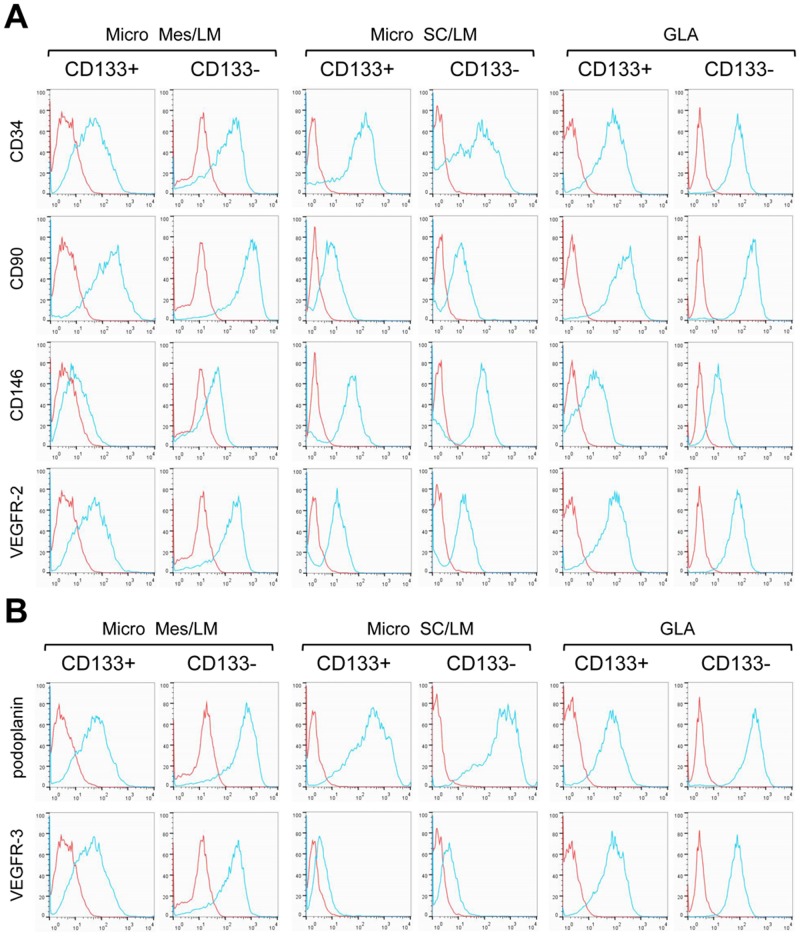
Endothelial precursor and lymphatic endothelial protein expression in isolated CD133^+^ and CD133^−^ LM cells. FACS of patient-matched CD133^+^ and CD133^−^ LM cells isolated from microcystic mesenteric (Micro Mes) LM, microcystic subcutaneous (Micro SC) LM and general lymphatic anomaly (GLA) specimens. (A) Endothelial precursor markers, CD34, CD90, CD146 and VEGFR-2. (B) Lymphatic endothelial cell markers, podoplanin and VEGFR-3. Blue line represents antibody data, and red line IgG control.

CD133^+^ cells were isolated from resected LM tissues and fluids, and compared to CD133-negative (CD133^−^) cells isolated from LM fluids. LMs specimens included cervicofacial (CF) LMs, mesenteric (Mes) LMs, subcutaneous (SC) LMs and GLAs. Patient-matched CD133^+^ and CD133^−^ LM cell populations were screened for the expression of markers of bone marrow and circulating endothelial precursors (CD34, CD90, CD146 and VEGFR-2), and for lymphatic endothelial markers (podoplanin and VEGFR-3) [14, 21]. Both CD133^+^ and CD133^−^ LM cells expressed CD34, CD90, and VEGFR-2 (Fig. 2). CD133^+^ LM cells also expressed CD146, podoplanin, and VEGFR-3, and their expression was often increased in the patient-matched CD133^−^ LM cells.

In murine inflammatory and tumor lymphangiogenesis, a subset of LECs has been suggested to arise from a myeloid/monocyte cell [14, 22, 23]. The expression of the myeloid marker, CD45, and the monocyte marker, CD11b, was assessed in CD133^+^ and CD133^−^ LM cells. CD11b expression was absent or low in a majority of the podoplanin^+^ cells, irrespective of their CD133 status (Fig. IIA in S2 File). CD45 expression was not observed (data not shown).

We assessed transcript expression of the stem cell markers, Oct4, NANOG and Sox2, as well as the endothelial precursor marker, c-Kit. Expression of c-Kit, Oct4, and NANOG was detected in CD133^+^ LM cells, and significantly lower or absent in CD133^−^ LM cells (Fig. IIB in S2 File). Sox2 was not expressed in either LM cell population (data not shown).

In summary, CD133^+^ cells isolated from LM patient samples expressed markers of endothelial precursors (CD34, CD90, CD146, VEGFR-2) and LECs (podoplanin, VEGFR-3), as well as stem cells. As compared to CD133^+^ cells, CD133^−^ LM cells had similar expression levels of endothelial precursor markers (CD90, CD146, VEGFR-2), but had increased expression of the lymphatic endothelial specific markers, (podoplanin, VEGFR-3) and lower expression of stem cell genes. Thus, CD133^+^ and CD133^−^ cells isolated from LM specimens are related, but distinct. CD133^+^ LM cells were more progenitor-like, while CD133^−^ LM cells were similar to mature LECs.

### CD133^−^ LM cells exhibit features consistent with lymphatic endothelial cells

**Fig 3:**
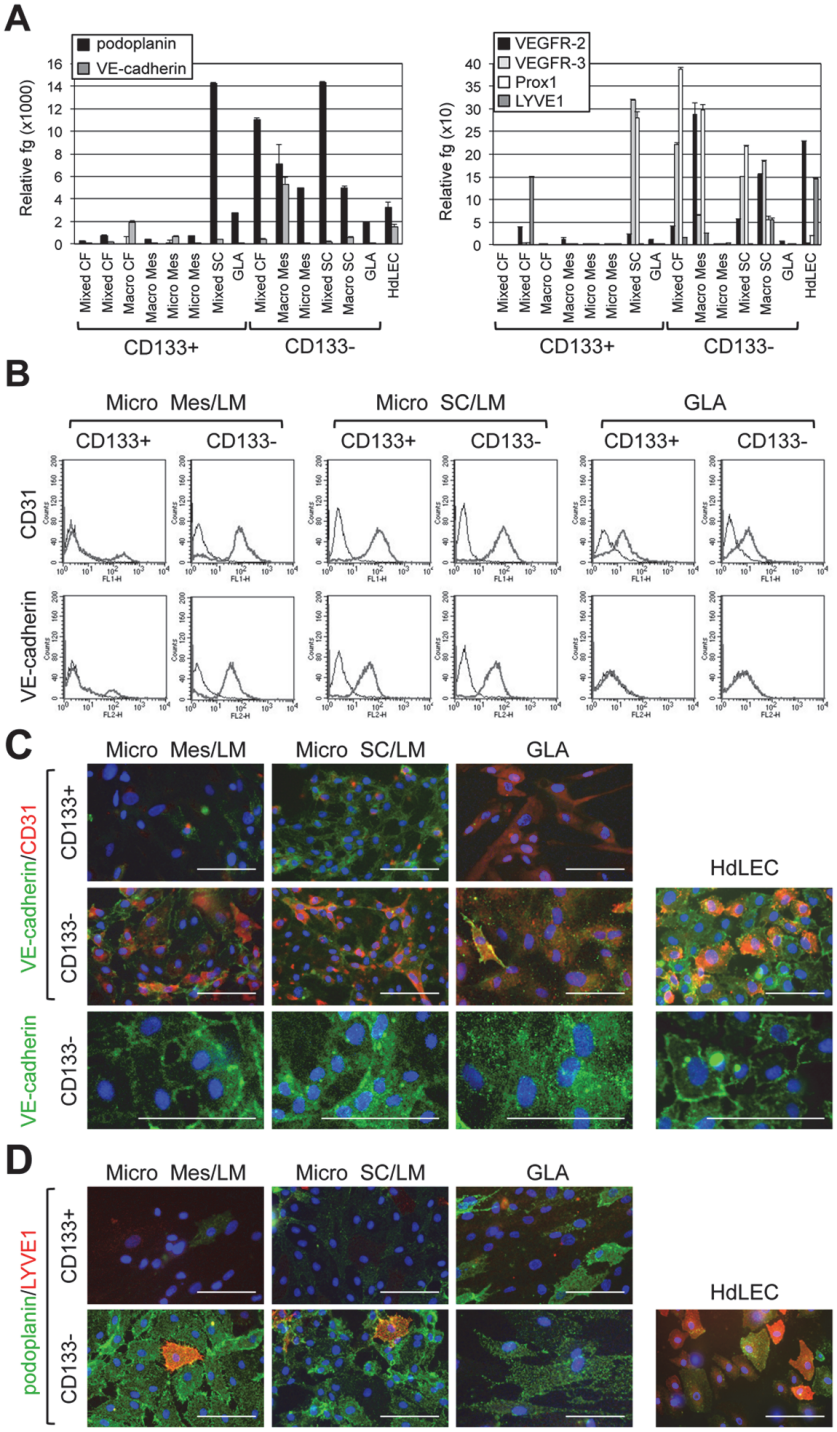
Expression of markers for mature lymphatic endothelial cells in isolated CD133^+^ and CD133^−^ LM cells. (A) Podoplanin, VE-cadherin, VEGFR-2, VEGFR-3, Prox1, and LYVE1 qRT-PCR of RNA isolated from CD133^+^ and CD133^−^ cells from LMs of different subtypes and anatomical locations and GLA compared to control HdLECs. Data normalized to β-actin qRT-PCR and represented as mean ± s.e.m. (B) CD31 and VE-cadherin FACS of patient-matched CD133^+^ and CD133^−^ LM cells isolated from microcystic mesenteric (Micro Mes) LM, microcystic subcutaneous (Micro SC) LM and general lymphatic anomaly (GLA) specimens. Thick gray line represents antibody data, and black line IgG control. (C) VE-cadherin/CD31 and (D) podoplanin/LYVE1 staining of patient-matched CD133^+^ and CD133^−^ LM cells isolated from microcystic mesenteric (Micro Mes) LM, microcystic subcutaneous (Micro SC) LM, and GLA compared to control HdLEC. Scale bars: 50μm.

CD133^−^ cells isolated from fluids collected from 15 LM patients (cervicofacial LMs (n = 4), mesenteric LMs (n = 4), thoracic LM (n = 1), subcutaneous LMs (n = 4) and GLA (n = 2)) were compared to CD133^+^ LM cells and neonatal human dermal lymphatic endothelial cells (HdLEC; n = 3). Expression of endothelial (VE-cadherin, VEGFR-2) and lymphatic endothelial (podoplanin, VEGFR-3, Prox1, LYVE1) gene transcripts was determined (Fig. 3A). As compared to CD133^+^ LM cells that expressed low levels of endothelial and LEC genes, a majority of CD133^−^ LM cells expressed higher levels of VE-cadherin, VEGFR-2, VEGFR-3, and Prox1. Podoplanin, Prox1 and VEGFR-3 expression levels were often higher than that those observed for HdLECs. In contrast, CD133^−^ LM cells expressed lower levels of LYVE1 than HdLECs. Two of the CD133^−^ LM cell populations, one isolated from a microcystic mesenteric LM and the other isolated from a GLA specimen, only expressed podoplanin similar to HdLECs, whereas VEGFR-2, VEGFR-3, Prox1 and LYVE1 expression was more similar to that of CD133^+^ LM cells. This expression pattern is consistent with these two CD133^−^ LM cell populations being relatively less differentiated.

FACS and immunostaining were performed on patient-matched CD133^+^ and CD133^−^ LM cells to assess expression of the mature endothelial markers, VE-cadherin and CD31. All CD133^−^ LM cells expressed CD31, where as its expression was inconsistently observed in CD133^+^ LM cells (Fig. 3B, 3C). Although VE-cadherin expression was observed in majority of CD133^−^ LM cells by immunostaining, its expression was poorly localized to adherens junctions as compared to HdLECs (Figs. 3C bottom panels). Consistent with the immunostaining of the CD133^−^ LM cells isolated from a GLA, FACS for VE-cadherin did not detect cell surface expression (Figs. 3B, 3C).

Patient-matched CD133^+^ and CD133^−^ LM cells were immunostained for the lymphatic endothelial markers, LYVE1 and podoplanin. Expression of podoplanin was higher in CD133^−^ LM cells relative to CD133^+^ LM cells (Fig. 3D). When compared to HdLECs, CD133^−^ LM cells expressed lower levels of LYVE1 and higher levels of podoplanin (Fig. 3D), similar to the abnormal lymphatic endothelium in LM tissues (Fig. 1A) and the transcriptional analyses (Fig. 3A).

Overall, expression of LEC genes was lower in CD133^+^ LM cells relative to CD133^−^ cells, whereas CD133^−^ LMs were more similar to HdLECs. Based on our assessment of markers, we designated CD133^+^ LM cells as lymphatic malformation progenitor cells (LMPCs), and CD133^−^ LM cells as lymphatic malformation endothelial cells (LMECs).

### LMPCs are multipotent and can be induced to differentiate down multiple lineages

**Fig 4:**
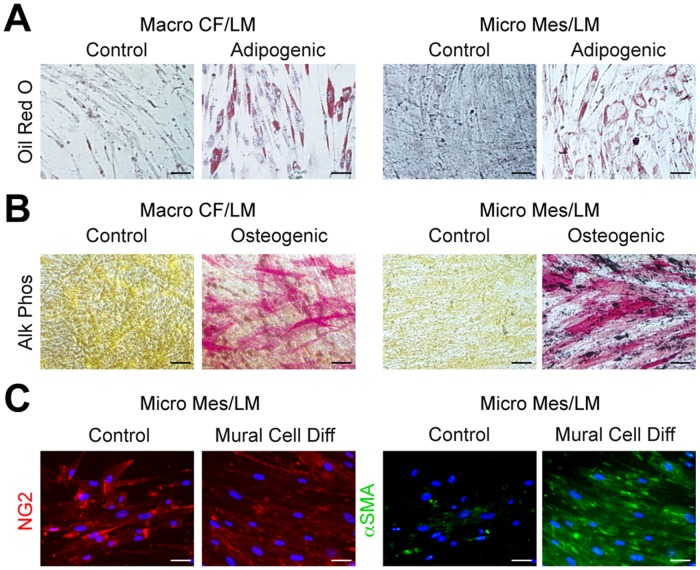
Differentiation of LMPCs into fat, bone and smooth muscle cells. (A) Oil Red O staining of LMPCs isolated from macrocystic cervicofacial (Macro CF) LM and microcystic mesenteric (Micro Mes) LM after 2 weeks in growth media (control) or adipogenic media. (B) Alkaline phosphatase (Alk Phos) staining of LMPCs isolated from macrocystic Macro CF LM and Micro Mes LM after 2 weeks in growth media (control) or osteogenic media. (C) NG2 and alpha smooth muscle actin (αSMA) staining of LMPCs isolated from Micro Mes LM after 2 weeks in growth media (control) or mural cell differentiation (Diff) media. Scale bars: 50μm.

We hypothesized that LMPCs are progenitor-like and predicted that they would be multipotent. We thus assessed the ability of CD133^+^ LMPCs to be induced down multiple cell lineages. 5 LMPC populations and 3 LMEC populations were cultured in induction media for adipocytes, osteoblasts, and vascular smooth muscle cells (VSMCs). After two weeks in adipogenic medium, LMPCs, but not LMECs, differentiated into adipocytes as assessed by Oil Red O staining for lipid accumulation (Fig. 4A, Fig. IIIA in S2 File). Similarly, only LMPCs differentiated into osteoblasts, as demonstrated by the presence of alkaline phosphatase activity (Fig. 4B, Fig. IIIB in S2 File). LMPC adipogenesis and osteogenesis was similar to that observed for human MSCs (Fig. III in S2 File).

LMPCs cultured in mural cell differentiation media for two weeks expressed alpha smooth muscle cell actin (αSMA), consistent with VSMC differentiation (Fig. 4C). LMPCs maintained in growth medium expressed higher levels of the pericyte marker, NG2, than those in the mural cell differentiation media (Fig. 4C). We assessed whether NG2 expression was observed in LM tissues and found that CD133^+^ cells co-expressed NG2 (Fig. IVA in S2 File). Cultured CD133^+^ LMPC populations expressed NG2, while NG2 expression was lower in patient-matched CD133^−^ LMECs (Figs. IVB, S4C in S2 File). Thus, NG2 appears to be a marker of LMPCs and its expression was lower in LMECs relative to LMPCs.

FACS analyses indicated that LMPCs are a heterogeneous population of cells (Fig. 2). To determine if CD34^+^ or podoplanin^+^ LMPCs are multipotent, three early passage LMPC populations were live cell sorted for CD34 and podoplanin, and positive and negative populations collected. Sorted LMPCs were cultured in adipogenic, osteogenic and mural cell differentiation media for 2 weeks and cell differentiation assessed. CD34^+^ and podoplanin^+^ LMPCs differentiated into fat, bone and VSMCs (Figs. VA-C in S2 File). CD34^−^ or podoplanin^−^ cells from two LMPC populations failed to expand after FACS. CD34^−^ or podoplanin^−^ cells isolated from a subcutaneous LM did differentiate down the bone pathway, but poorly differentiated down fat or VSMC pathways (data not shown). Thus, multipotency was observed in the CD34^+^ and podoplanin^+^ LMPC populations.

### LMPCs differentiate into LMECs in vitro

**Fig 5:**
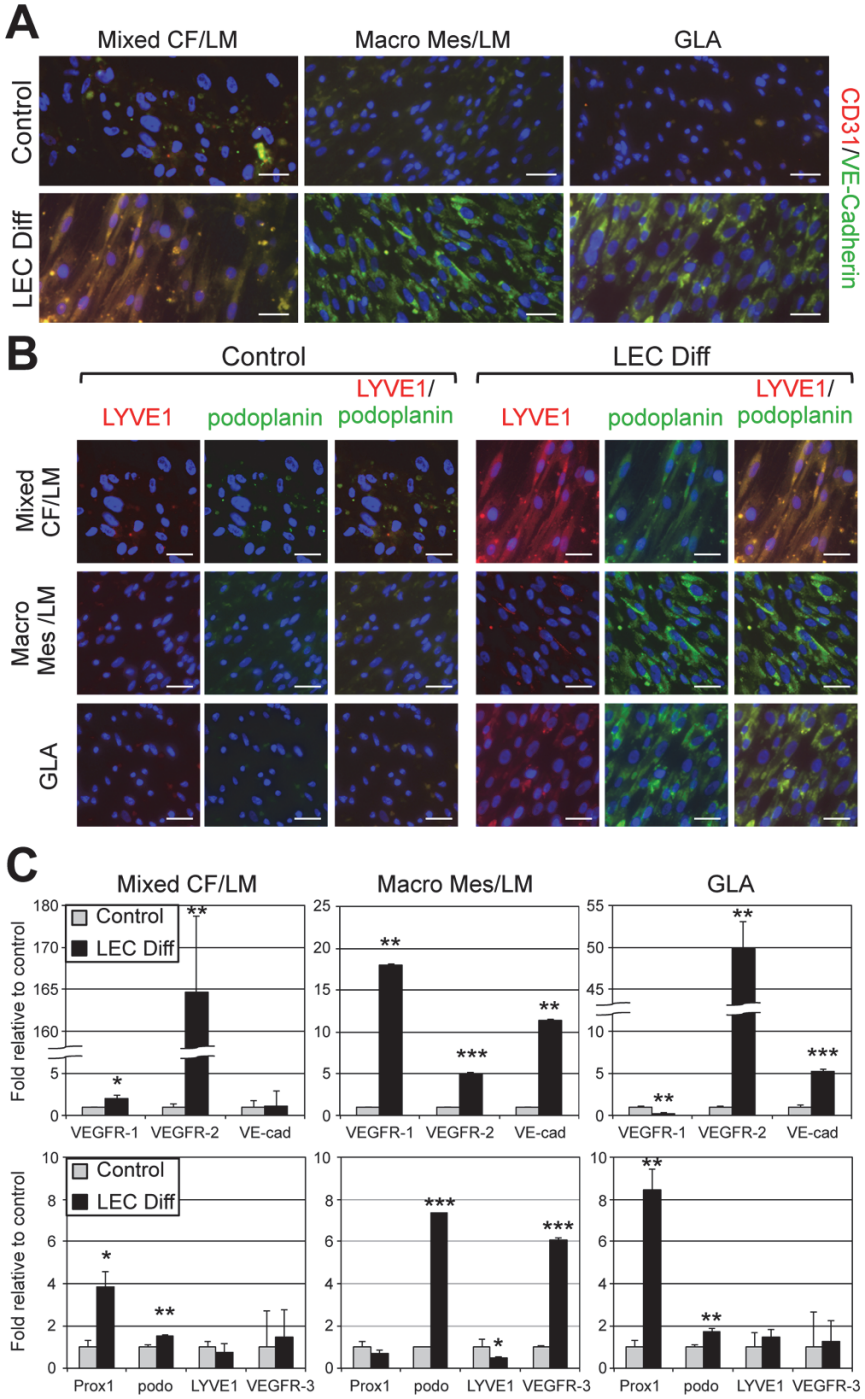
LMPCs differentiated into abnormal lymphatic endothelial cells. LMPCs isolated from mixed cervicofacial (Mixed CF) LM, macrocystic mesenteric (Macro Mes) LM and a generalized lymphatic anomaly (GLA) were maintained in growth media (control) or lymphatic endothelial differentiation (LEC Diff) media for two weeks. (A) CD31/VE-cadherin and (B) LYVE1/podoplanin staining. Scale bars: 50μm. (C) VEGFR-1, VEGFR-2, VE-cadherin, Prox1, Podoplanin, LYVE1, and VEGFR-3 qRT-PCR of RNA isolated from LMPCs maintained in growth or LEC differentiation (LEC Diff) media. Data normalized to β-actin qRT-PCR and represented as mean ± s.e.m. * p < 0.05, ** p < 0.005, *** p < 0.0005.

To determine if LMPCs could differentiate into LMECs, we cultured five LMPC populations and three CD34^+^ or podoplanin^+^ LMPC populations in LEC differentiation media. After two weeks, LMPCs strongly expressed VE-cadherin and podoplanin, while CD31 and LYVE1 expression were modestly upregulated (Figs. 5A, 5B; Fig. VD in S2 File). In contrast, expression of all four endothelial and lymphatic endothelial proteins was low in LMPCs maintained in growth media. In LMPCs grown in LEC differentiation media, VE-cadherin was cytoplasmic and not localized to the adherens junctions, and LYVE1 was poorly expressed, similar to LMECs (Figs. 3C, 5A, 5B). In all LMPCs induced down the LEC fate, podoplanin and VEGFR-2 transcripts were significantly upregulated, but LYVE1 was not induced (Fig. 5C). Similar to the heterogeneity observed in the different LMEC populations, LEC markers, VE-cadherin, Prox1, and VEGFR-3 were only upregulated in a subset of the LMPCs in differentiation media. Although not normally expressed by LECs, the blood endothelial cell-specific VEGFR-1 was increased in two of the LMPCs induced down the lymphatic endothelial pathway. LMPCs differentiated into LECs that appear similar to CD133^−^ LMECs isolated from patients.

### LMPCs recapitulate the LM phenotype in mice

**Fig 6:**
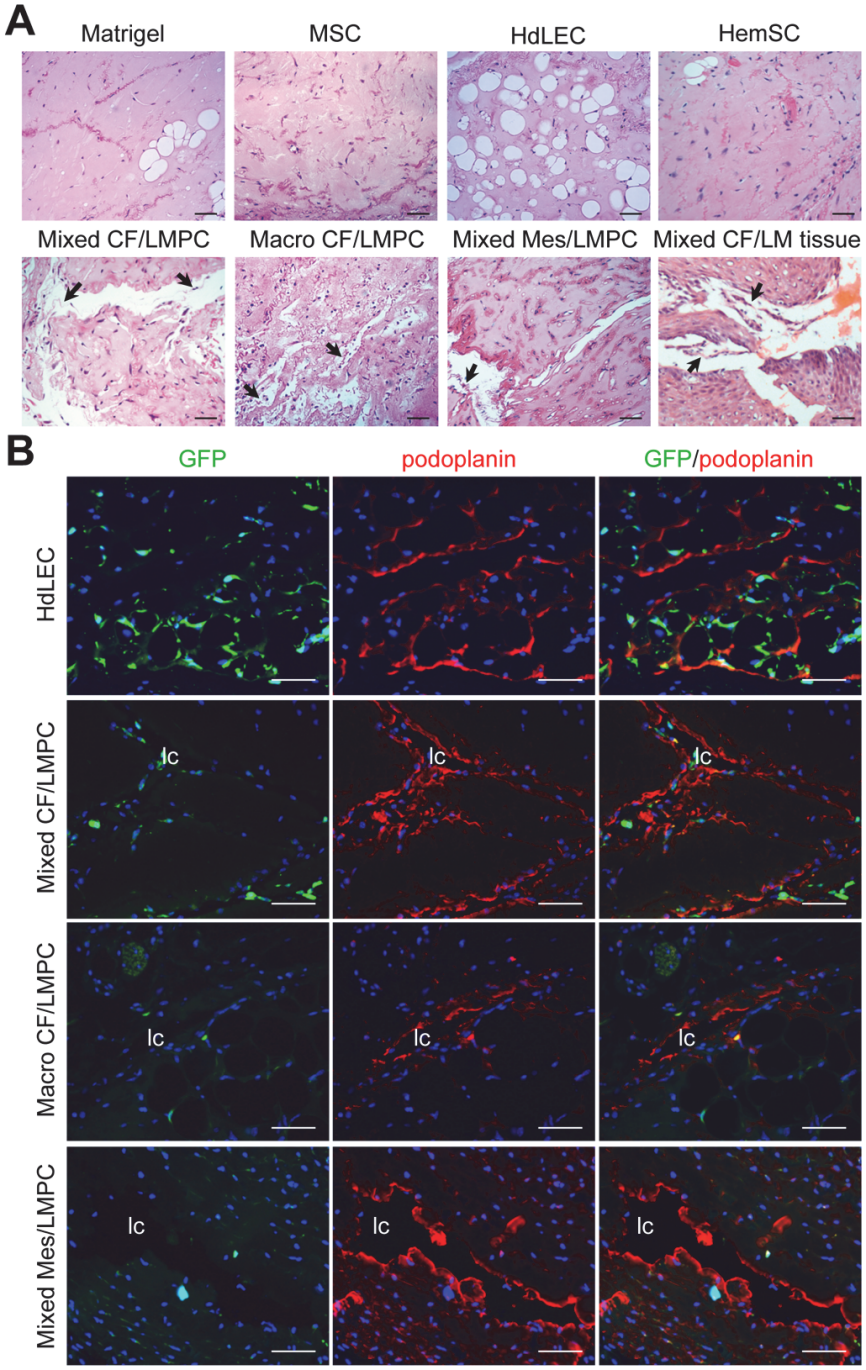
LMPC recapitulated the LM phenotype in a mouse model. LMPCs isolated from mixed cervicofacial (Mixed CF), macrocystic cervicofacial (Macro CF) and mixed mesenteric (Mixed Mes) LM were suspended in Matrigel and implanted into GFP-expressing immunocompromised mice. Matrigel alone, mesenchymal stem cells (MSCs), normal human dermal lymphatic endothelial cells (HdLEC) and hemangioma stem cells (HemSC) served as controls. (A) Hematoxylin and eosin (H&E) staining of xenograft sections. (B) GFP (host cell) and podoplanin staining of xenograft sections. Scale bars: 50μm. lymphatic channel (lc)

To determine whether isolated LMPCs have the capacity to form lesions *in vivo* that phenocopy human LMs, six CD133^+^ LMPC populations isolated from cervicofacial (CF, n = 2), mesenteric (Mes, n = 2), and subcutaneous (SC, n = 1) LMs, and a GLA were suspended in Matrigel and implanted into GFP-expressing immunocompromised mice. Matrigel implanted alone or Matrigel with normal neonatal HdLECs or MSCs served as controls. The proposed progenitor cells of infantile hemangioma, HemSCs (hemangioma stem cells), were also assessed in the xenograft model. Implants were removed at 3 and 5 weeks, and sections were H&E stained. Similar to patient LM tissue, large channels and ectatic lymphatic vessels were observed in the LMPC implants (Fig. 6A). In both LMPC implants and LM patient tissues, sloughing of the endothelium into the dilated lymphatic channels was observed. Unlike LMPCs isolated from lymphatic anomalies, HemSCs from a blood vessel anomaly, infantile hemangioma [11], did not develop dilated channels. Aberrant vessels were not observed in Matrigel implants, or implants that contained MSC or HdLEC.

LMPC and control implants were immunostained for GFP to identify host-derived cells, and human-specific podoplanin to visualize LM-cell derived lymphatic vessels. In HdLEC implants, lymphatic vessels stained strongly for podoplanin and were observed to lie between host-derived GFP^+^ adipocytes (Fig. 6B). In LMPC implants, the cells lining the dilated channels stained positive for podoplanin and negative for GFP, demonstrating that the abnormal lymphatic vessels were derived from the LMPCs (Fig. 6B). Podoplanin expression was not observed in MSC or Matrigel controls (Fig. VI in S2 File).

**Fig 7:**
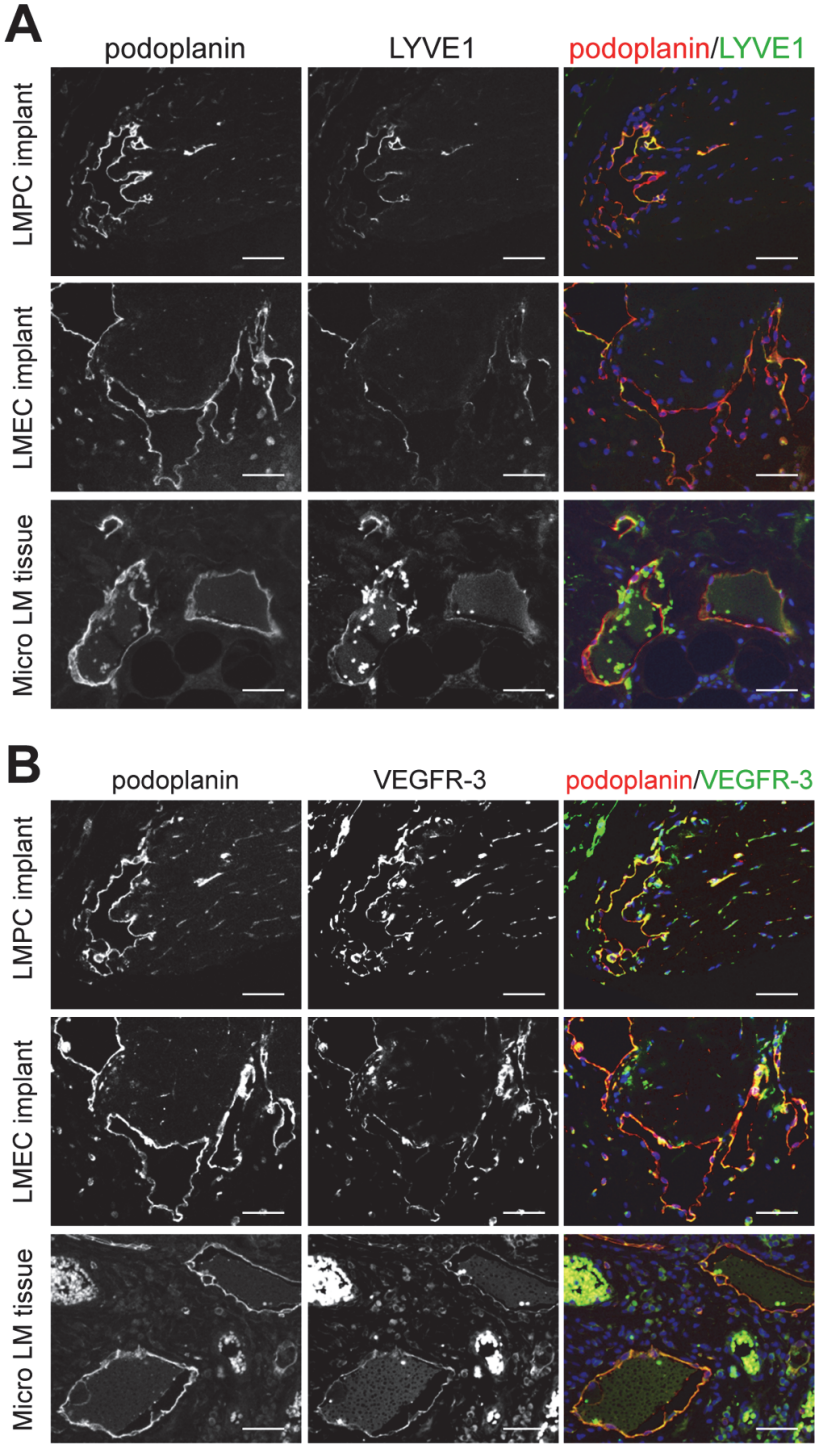
Patient-matched LMPC and LMEC implants expressed the lymphatic proteins, LYVE1 and VEGFR-3. CD133+ LMPCs and CD133- LMECs isolated from a microcystic subcutaneous LM were suspended in Matrigel and implanted in immunocompromised mice. Staining of implants was compared to microcystic subcutaneous LM patient tissue (Micro LM tissue). (A) Podoplanin and LYVE1 and (B) podoplanin and VEGFR-3 staining. Scale bars: 50μm.

**Fig 8:**
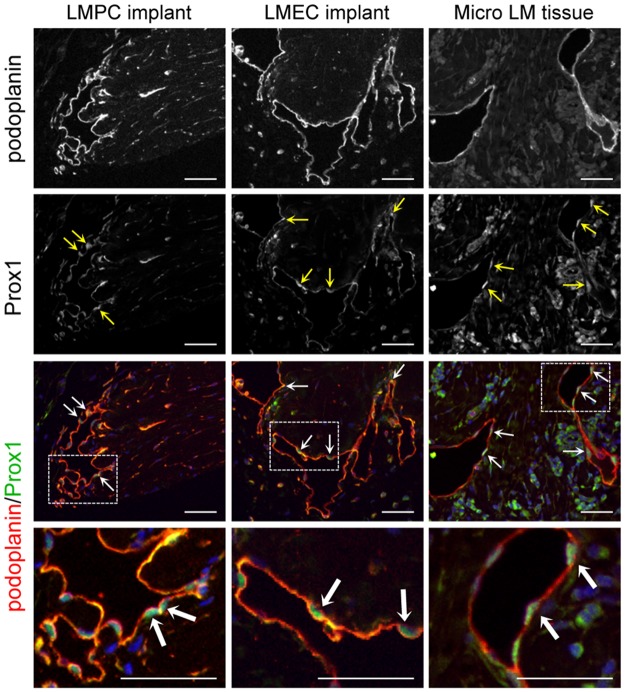
Patient-matched LMPC and LMEC implants expressed the lymphatic endothelial master regulator, Prox1. CD133^+^ LMPCs and CD133^−^ LMECs isolated from a microcystic subcutaneous LM were suspended in Matrigel and implanted in immunocompromised mice. Podoplanin and Prox1 staining of implants was compared to microcystic subcutaneous LM patient tissue (Micro LM tissues). Arrows mark Prox1 positive nuclei. Boxed areas enlarged below. Scale bars: 50μm.

To further characterize the podoplanin^+^ vessels in the LMPC implants, patient-matched CD133^+^ LMPC and CD133^−^ LMEC implants were stained for additional lymphatic endothelial specific proteins, LYVE1, Prox1 and VEGFR-3. Similar to patient LM tissues, expression of LYVE1 was variable in the cells lining the dilated lymphatic vessels of LMPC and LMEC implants (Fig. 7A). The aberrant lymphatic vessels in LMPC and LMEC implants also stained positive for Prox1 and VEGFR-3 (Figs. 7B, 8). The expression levels and patterns of podoplanin, LYVE1, Prox1 and VEGFR-3 were similar in the dilated vessels of LMPC and LMEC implants. In the xenograft model, LMPCs differentiated into LMECs and recapitulated the LM phenotype.

## Discussion

We have identified and isolated lymphatic malformation progenitor cells from LM patients. CD133^+^/podoplanin^+^ LMPCs were observed in the abnormal lymphatic endothelium and adjacent parenchyma of LM tissues, independent of anatomic location (cervicofacial, mesenteric, thoracic, subcutaneous, diffuse) or sub-type (macrocystic, microcystic, mixed macro/microcystic, GLA), suggesting that LMPCs are common to lymphatic anomalies. Isolated CD133^+^ LMPCs expressed multiple markers of stem cells (NANOG, Oct4), endothelial cell precursors (CD34, CD90, CD146, c-Kit, VEGFR-2) and lymphatic endothelial proteins (podoplanin, VEGFR-3). *In vitro*, LMPCs were multipotent, and could be induced into adipocytes, osteocytes, smooth muscle cells and LECs. Characterization of CD133^−^ LM cells isolated from LM fluids indicated these cells were lymphatic malformation endothelial cells. Relative to CD133^+^ LMPCs, CD133^−^ LMECs had decreased or absent expression of stem cell genes (NANOG, Oct4, c-Kit), maintained the expression of the endothelial precursor markers (CD90, CD146, VEGFR-2), and exhibited increased expression of the endothelial/lymphatic endothelial markers (VE-cadherin, CD31, podoplanin, VEGFR-3, Prox1). In addition, CD133^−^ LMECs were not multipotent. Finally, CD133^+^ LMPC xenografts developed abnormal lymphatic vessels that expressed podoplanin, LYVE1, Prox1 and VEGFR-3, similar to patterns observed in LM patient tissues and CD133^−^ LMEC xenografts. Taken together, this data provides strong evidence that LMPCs are the cell type of origin in LMs.

In humans, CD133^+^/CD34^+^/VEGFR-3^+^ cells isolated from the fetal liver have been shown to differentiate into LECs *in vitro* [21]. These CD133^+^/CD34^+^/VEGFR-3^+^ LEC progenitors were enriched in the fetal liver, but also present in fetal cord blood. In adult mice, bone marrow-derived progenitors have been shown to incorporate into the lymphatic endothelium in mouse models of inflammation-induced lymphangiogenesis in the cornea, wound healing, and tumor xenografts [14, 22–24]. These bone marrow-derived LEC progenitors have been described as either CD34^+^/VEGFR-2^+^/VEGFR-3^+^ [24] or podoplanin^+^/c-Kit^+^/VEGFR-2^+^/CD11b^+^ [14]. The CD133^+^ cells we isolated from LM specimens expressed multiple markers of fetal and adult LEC progenitors, including CD133, CD34, podoplanin, c-Kit, VEGFR-2 and VEGFR-3. LMPCs did not express high levels of CD11b, but did expressed CD90, CD146 and NG2. Bone marrow MSCs have been described to be express CD90, CD146, and NG2 [25–28], while neuronal progenitors also express CD90 and NG2 [26, 29, 30]. Taken together, these data suggest that LMPCs are abnormal lymphatic endothelial progenitors that likely arise from the bone marrow or a neuronal progenitor. Alternatively, LMPCs may be derived from a multipotent pericyte progenitor [31, 32]. Similar to LMPCs, pericytes express CD90, CD146 and NG2 and can be induced to differentiate down the bone, fat, and cartilage pathways [33]. However, pericytes are defined as negative for the endothelial precursor marker, CD34, which is expressed by LMPCs [32, 33]. Thus, the cell-type or organ of origin for LMPCs remains undefined.

Podoplanin^+^/VEGFR-3^+^/CD31^+^ LECs isolated from LM tissues have previously been shown to develop abnormal podoplanin^+^/LYVE1^low^ lymphatics in Matrigel implants in mice [34]. We demonstrate that CD133^+^ LMPCs that are podoplanin^+^/VEGFR-3^+^/CD31^−^, as well as CD133^−^ LMECs that are podoplanin^+^/VEGFR-3^+^/CD31^+^, develop ectatic aberrant lymphatics that phenocopy the lymphatic vessel morphology in LM tissues. Like LMEC implants and LM tissues, lymphatic vessels in LMPC implants express podoplanin, VEGFR-3, and Prox1, and discontinuous and low levels of LYVE1. Thus, we propose that CD133^+^ LMPCs contribute to the pathological lymphatic vessel development of LMs.

The abnormal dilated lymphatic vessels in LM patients and LMPC implants shared some similarities with the immature lymphatic vessels in the neonatal dermis. Unlike mature lymphatics in uninvolved tissues, the immature lymphatic vessels in neonatal tissue expressed high levels of CD133, similar to LM vessels. In contrast, LYVE1 expression was discontinuous and reduced in the aberrant lymphatic vessels in LM tissues and LMPC or LMEC xenografts. Low-level LYVE1 expression was also observed in LMECs isolated from patients or differentiated from LMPCs *in vitro*. LYVE1 function in the lymphatic vasculature is poorly understood, and embryonic lymphatic vessel growth appears unaffected in *LYVE1* knockout mice [35]. However, closer analysis of the vessel morphology of adult mice has demonstrated that *LYVE1* knockout mice have distended or disorganized lymphatics in the intestines and liver [36]. LYVE1 was recently shown to bind FGF-2 and function to suppress FGF-2-induced LEC proliferation, migration, and invasion [37]. Thus, the defect in LYVE1 expressions observed in LMECs may contribute to the abnormal dilated lymphatic phenotype observed in LM lesions.

Unlike HdLECs that formed uniform and nondilated lymphatics in the mouse model, LMPCs differentiated into LMECs that formed ectatic lymphatic vessels, with sloughing of the abnormal lymphatic endothelium into vessel lumens. This sloughing may be due to defective LEC-LEC interactions. Consistent with this notion, LMECs were abundant in the LM patient fluid aspirates. We posit that this LMEC shedding may be secondary to defects in VE-cadherin function. Although VE-cadherin was expressed in LMECs or LMEC differentiated from LMPCs, it was often not present in adherens junctions. This improper localization of VE-cadherin may be secondary to defects in other components of the adherens junctions, such as α- or β-catenin.

Patients with well-circumscribed macrocystic LMs can often be effectively treated by surgical excision of the affected area, or by sclerotherapy. However, a majority of LMs present with wholly or partially microcystic or diffuse disease that responds poorly to surgery and sclerotherapy, with recurrence observed in 22–59% of LM patients [5, 6]. The presence of lymphatic progenitor cells, after surgical or ablative chemical treatment, may explain this high rate of recurrence. Thus, therapeutic interventions that target both the abnormal lymphatic endothelium and the progenitor cell population in LMs may be required for clinical efficacy. We propose that identification of LMPCs and the development of a mouse LM model that we describe here will provide critical tools to allow for development of treatment options for patients with LMs.

## Supporting Information

S1 File File includes Tables I-III. Table I: Summary of LM tissues and cells analyzed. Table II: Antibodies. Table III: Quantitative RT-PCR Primers.
(PDF)

S2 File File includes Figs. I-VI. Fig. I: LM tissues histology. (A) H&E staining of patient-matched uninvolved and mixed cervicofacial (Mixed CF) LM tissues. (B) H&E staining of neonatal control tissue, Mixed CF LM or Gorham’s tissues. Arrowheads mark abnormal lymphatic vessels. Scale bars: 50μm. lymphatic channel (lc). Fig. II: Analysis of progenitor and stem cell markers in CD133^+^ and CD133^−^ LM cells. (A) CD11b and podoplanin FACS of patient-matched CD133^+^ and CD133^−^ LM cells isolated from microcystic mesenteric (Micro Mes) LM, microcystic subcutaneous (Micro SC) LM and general lymphatic anomaly (GLA) specimens. (B) NANOG, Oct4 and c-Kit qRT-PCR of RNA isolated from patient-matched CD133^+^ and CD133^−^ LM cells isolated from a macrocystic mesenteric LM. Data normalized to β-actin qRT-PCR and represented as mean ± s.e.m. * p < 0.01, ** p < 0.0005. Fig. III: LMPCs and not LMECs were multipotent. (A) Oil Red O staining of MSCs, LMPCs isolated from mixed cervicofacial (Mixed CF) LM and patient-matched LMPCs and LMECs isolated from macrocystic mesenteric (Macro Mes) LM after 2 weeks in adipogenic media. (B) Alkaline phosphatase staining of MSC, LMPCs isolated from Mixed CF LM and patient matched LMPCs and LMECs isolated from Macro Mes LM after 2 weeks in osteogenic media. Scale bars: 50μm. Fig. IV: LMPCs expressed NG2. (A) NG2 and CD133 staining of mixed cervicofacial (Mixed CF) and microcystic mesenteric (Micro Mes) LM tissues. White arrowheads mark abnormal lymphatic vessels. (B) NG2 staining of CD133^+^ LM cells isolated from Mixed CF LM, Micro Mes LM tissues and generalized lymphatic anomaly (GLA) specimens. (C) NG2 staining of patient-matched CD133^+^ LMPCs and CD133^−^ LMECs isolated from macrocystic mesenteric (Macro Mes) LM and GLA. Scale bars: 50μm. lymphatic channel (lc). Fig. V: CD34+ or podoplanin+ LMPCs were multipotent. LMPCs isolated from a microcystic subcutaneous were sorted for CD34 or podoplanin positivity and induced to differentiate into fat, bone, VSMCs and LECs. (A) Oil Red O staining of CD34^+^ or podoplanin^+^ LMPCs after 2 weeks in growth media (control) or adipogenic media. (B) Alkaline phosphatase (Alk Phos) staining of CD34^+^ or podoplanin^+^ LMPCs after 2 weeks in growth media (control) or osteogenic media. (C) Alpha smooth muscle actin (αSMA) staining of CD34^+^ or podoplanin^+^ LMPCs after 2 weeks in growth media (control) or mural cell differentiation (Diff) media. (D) Podoplanin and LYVE1 staining of CD34^+^ or podoplanin^+^ LMPCs after 2 weeks in growth media (control) or LEC differentiation (Diff) media. Scale bars: 50μm. Fig. VI: Analysis of control implants. Matrigel alone or MSCs suspended in Matrigel was implanted into GFP-expressing immunocompromised mice. GFP (host cell) and podoplanin staining of xenograft sections. Scale bars: 50μm.
(PDF)
