# Supplementary material for: Aberrant Lymphatic Endothelial Progenitors in Lymphatic Malformation Development
Source: PLoS One. 2015 Feb 26;10(2):e0117352. doi: 10.1371/journal.pone.0117352 (PMC4342011; doi:10.1371/journal.pone.0117352)
Supplement: S1 File — Table I: Summary of LM tissues and cells analyzed. Table II: Antibodies. Table III: Quantitative RT-PCR Primers. (PDF) [file pone.0117352.s001.pdf]

Table I. Summary of LM tissues and cells analyzed

No.	Subtype Location	IHC Tissue	IHC Cells	FACS	qRT-PCR	Differentiation Assays	LM Model
1	Mixed CF	X					
2	GLA	X					
3	Gorham's Stout	X					
4	Mixed CF	X	X	X	X	X	X
5	Mixed Limb	X					
6	Macro CF		X	X	X		
7	Macro SC	X	X	X	X	X	X
8	Macro Mes	X	X	X	X	X	X
9	GLA		X	X	X	X	X
10	Macro CF		X	X	X	X	X
11	Micro Mes		X	X	X	X	X
12	Macro SC		X	X	X	X	
13	GLA			X	X		
14	Micro SC	X					

CF – cervicofacial, GLA – generalized lymphatic anomaly, Macro – macrocystic LM, Mes – mesenteric LM, Micro – microcystic LM, Mixed - mixed micro/macrocystic LM, SC – subcutaneous, IHC-immunohistochemistry, FACS - Fluorescent-activated cell sorting, qRT-PCR – quantitative RT-PCR.

Table II. Antibodies

Antigen	Method	Company	Catalogue #
αSMA	IHC cells	Sigma	A2547
CD11b	FACS	BD Biosciences	562793 (FITC-conjugated)
CD133	Cell isolation	Miltenyi Biotec	130-097-049
	IHC tissue	Miltenyi Biotec	130-090-664
CD146	FACS	Millipore	MAB16985F (FITC-conjugated)
CD31	FACS	Ancell	180-040 (FITC-conjugated)
	IHC cells	abcam	ab32457
CD34	FACS	BD Biosciences	550619 (PE-conjugated)
CD90	FACS	BD Biosciences	555595 (FITC-conjugated)<br>555596 (PE-conjugated)
GFP	IHC tissue	Invitrogen	A-11122
LYVE1	IHC tissue<br>IH cells	R&D systems	AF2089
NG2	IHC tissue<br>IH cells	Millipore	AB5320
podoplanin	FACS	Angiobio	11-009PE (PE-conjugate)<br>17-9381-42 (APC-conjugated)
	IHC tissue<br>IHC cells	Angiobio	11-003
	IHC tissue	R&D systems	AF3670
Prox1	IHC tissue	R&D systems	AF2727
VE-cadherin	FACS	R&D systems	FAB9381P (PE-conjugated)
	IHC cells	abcam	ab7047
VEGFR-2	FACS	R&D Systems	FAB357P (PE-conjugated)
VEGFR-3	FACS	R&D Systems	FAB3492P (PE-conjugated)
	Tissue IHC	R&D Systems	AF349

Table III. Quantitative RT-PCR Primers

Gene	Upper Primer	Lower Primer
β-actin	5' CGAGGCCCAGAGCAAGAGAG 3'	5' CTCGTAGATGGGCACAGTGTG 3'
c-Kit	5' CTGGGATTTTCTCTGCGTTC 3'	5' GCCCACGCGGACTATTAAGT 3'
LYVE1	5' TAGCTTTGAAACTTGCAGCTATG 3'	5' TCAACAAATGGTTCAGTTTCTGTAG 3'
NANOG	5' GATTTGTGGGCCTGAAGAAA 3'	5' AAGTGGGTTGTTTGCCTTTG 3'
Oct4	5' GACAACAATGAGAACCTTCAGGAGA 3'	5' CTGGCGCCGGTTACAGAACCA 3'
Podoplanin	5' CCCAGGAGAGCAACAACTCAAC 3'	5' CTCGATGCGAATGCCTGTTAC 3'
Prox1	5' ACGTAAAGTTCAACAGATGCATTAC 3'	5' CCAGCTTGCAGATGACCTTG 3'
Sox2	5' GCGAACCATCTCTGTGGTCT 3'	5' GGAAAGTTGGGATCGAACAA 3'
VE-cadherin	5' TGTGACAGCAGTGGATGCAGA 3'	5' CTGTACTTGGTCATCCGGTTCTG 3'
VEGFR-1	5' GAAGGAGAGGACCTGAAACTGTC 3'	5' ACTCTTTCAATAAACAGCGTGCTG 3'
VEGFR-2	5' GGACTGGCTTTGGCCCAAT 3'	5' CTTGCTGTCCCAGGAAATTCTG 3'
VEGFR-3	5' GAGACCTGGCTGCTCGGAAC 3'	5' TCAGCATGATGCGGCGTATG 3'
