# Supplementary material for: Aberrant Lymphatic Endothelial Progenitors in Lymphatic Malformation Development
Source: PLoS One. 2015 Feb 26;10(2):e0117352. doi: 10.1371/journal.pone.0117352 (PMC4342011; doi:10.1371/journal.pone.0117352)
Supplement: S2 File — Fig. I: LM tissues histology. (A) H&E staining of patient-matched uninvolved and mixed cervicofacial (Mixed CF) LM tissues. (B) H&E staining of neonatal control tissue, Mixed CF LM or Gorham’s tissues. Arrowheads mark abnormal lymphatic vessels. Scale bars: 50μm. lymphatic channel (lc). Fig. II: Analysis of progenitor and stem cell markers in CD133+ and CD133− LM cells. (A) CD11b and podoplanin FACS of patient-matched CD133+ and CD133− LM cells isolated from microcystic mesenteric (Micro Mes) LM, microcystic subcutaneous (Micro SC) LM and general lymphatic anomaly (GLA) specimens. (B) NANOG, Oct4 and c-Kit qRT-PCR of RNA isolated from patient-matched CD133+ and CD133− LM cells isolated from a macrocystic mesenteric LM. Data normalized to β-actin qRT-PCR and represented as mean ± s.e.m. * p < 0.01, ** p < 0.0005. Fig. III: LMPCs and not LMECs were multipotent. (A) Oil Red O staining of MSCs, LMPCs isolated from mixed cervicofacial (Mixed CF) LM and patient-matched LMPCs and LMECs isolated from macrocystic mesenteric (Macro Mes) LM after 2 weeks in adipogenic media. (B) Alkaline phosphatase staining of MSC, LMPCs isolated from Mixed CF LM and patient matched LMPCs and LMECs isolated from Macro Mes LM after 2 weeks in osteogenic media. Scale bars: 50μm. Fig. IV: LMPCs expressed NG2. (A) NG2 and CD133 staining of mixed cervicofacial (Mixed CF) and microcystic mesenteric (Micro Mes) LM tissues. White arrowheads mark abnormal lymphatic vessels. (B) NG2 staining of CD133+ LM cells isolated from Mixed CF LM, Micro Mes LM tissues and generalized lymphatic anomaly (GLA) specimens. (C) NG2 staining of patient-matched CD133+ LMPCs and CD133− LMECs isolated from macrocystic mesenteric (Macro Mes) LM and GLA. Scale bars: 50μm. lymphatic channel (lc). Fig. V: CD34+ or podoplanin+ LMPCs were multipotent. LMPCs isolated from a microcystic subcutaneous were sorted for CD34 or podoplanin positivity and induced to differentiate into fat, bone, VSMCs and LECs. (A) Oil Red O staining of [file pone.0117352.s002.pdf]

Wu et al. Figure I

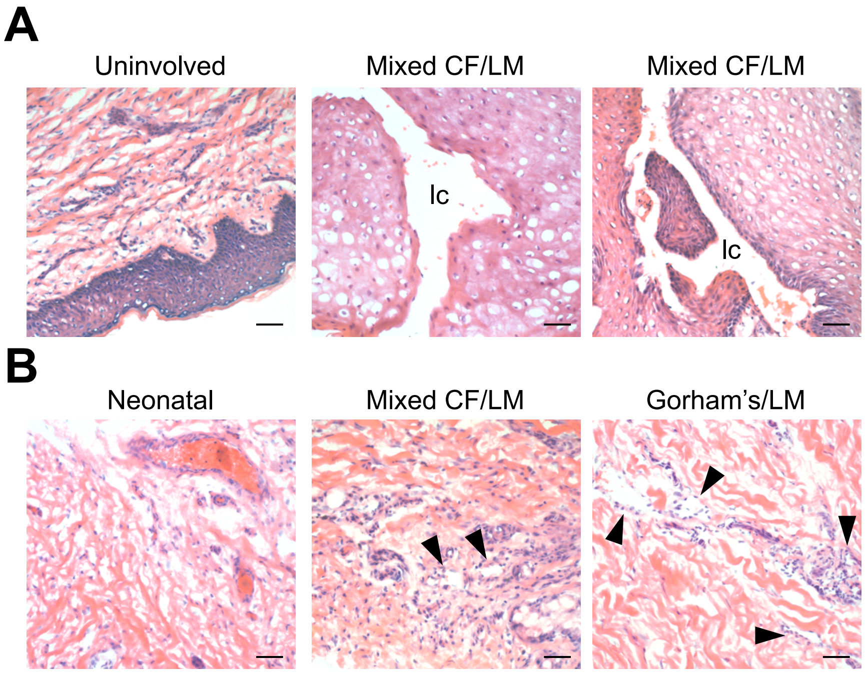

Figure S1. LM tissues histology. (**A**) H&E staining of patient-matched uninvolved and mixed cervicofacial (Mixed CF) LM tissues. (**B**) H&E staining of neonatal control tissue, Mixed CF LM or Gorham's tissues. Arrowheads mark abnormal lymphatic vessels. Scale bars: 50 μm. lymphatic channel (lc)

Wu et al. Figure II

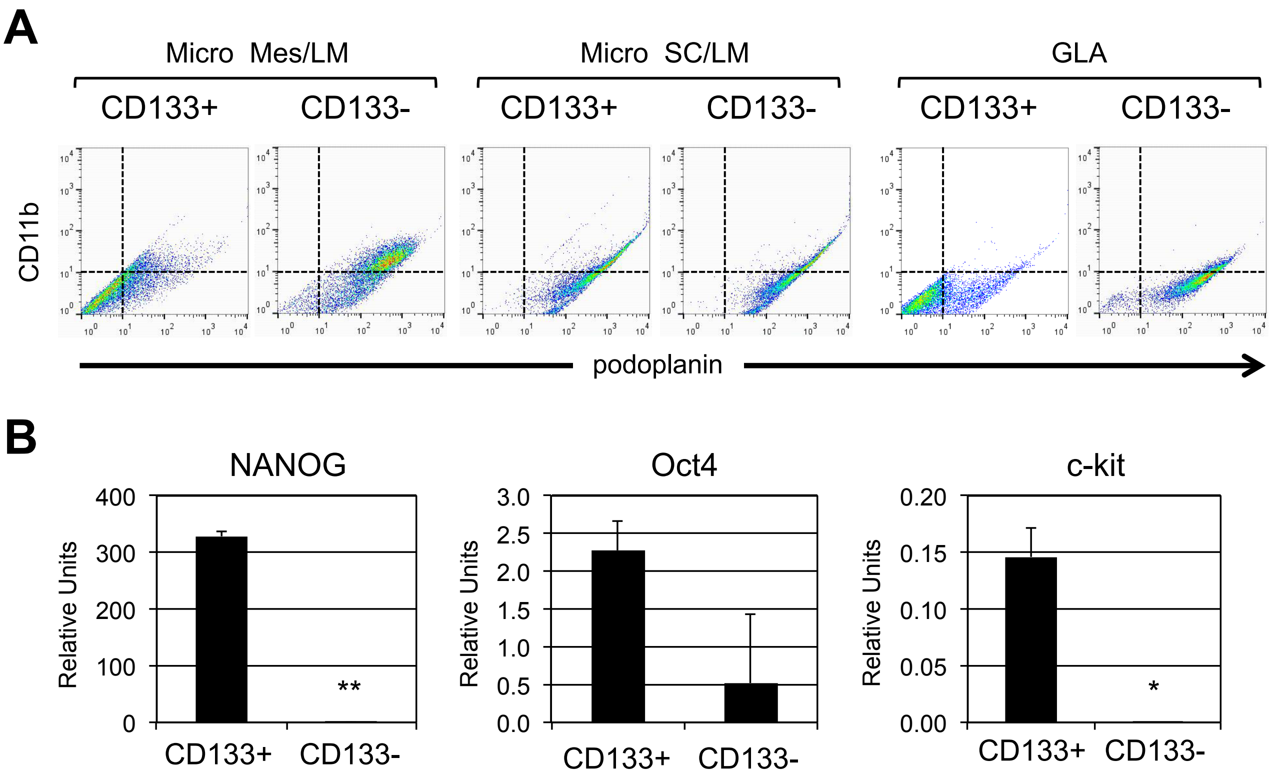

Figure S2. Analysis of progenitor and stem cell markers in CD133$^+$ and CD133$^-$ LM cells. (**A**) CD11b and podoplanin FACS of patient-matched CD133$^+$ and CD133$^-$ LM cells isolated from microcystic mesenteric (Micro Mes) LM, microcystic subcutaneous (Micro SC) LM and general lymphatic anomaly (GLA) specimens. (**B**) NANOG, Oct4 and c-Kit qRT-PCR of RNA isolated from patient-matched CD133$^+$ and CD133$^-$ LM cells isolated from a macrocystic mesenteric LM. Data normalized to β-actin qRT-PCR and represented as mean ± s.e.m. * $p < 0.01$, ** $p < 0.0005$.

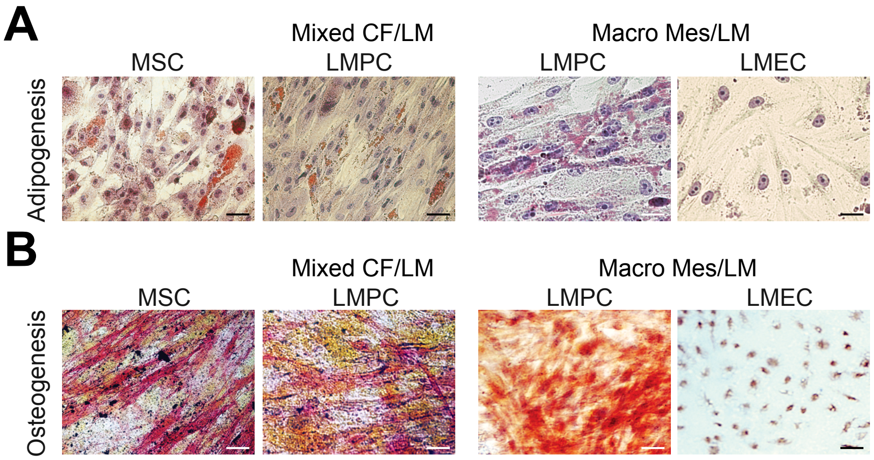

Figure S3. LMPCs and not LMECs were multipotent. (**A**) Oil Red O staining of MSCs, LMPCs isolated from mixed cervical facial (Mixed CF) LM and patient-matched LMPCs and LMECs isolated from macrocystic mesenteric (Macro Mes) LM after 2 weeks in adipogenic media. (**B**) Alkaline phosphatase staining of MSC, LMPCs isolated from Mixed CF LM and patient matched LMPCs and LMECs isolated from Macro Mes LM after 2 weeks in osteogenic media. Scale bars: 50 μm.

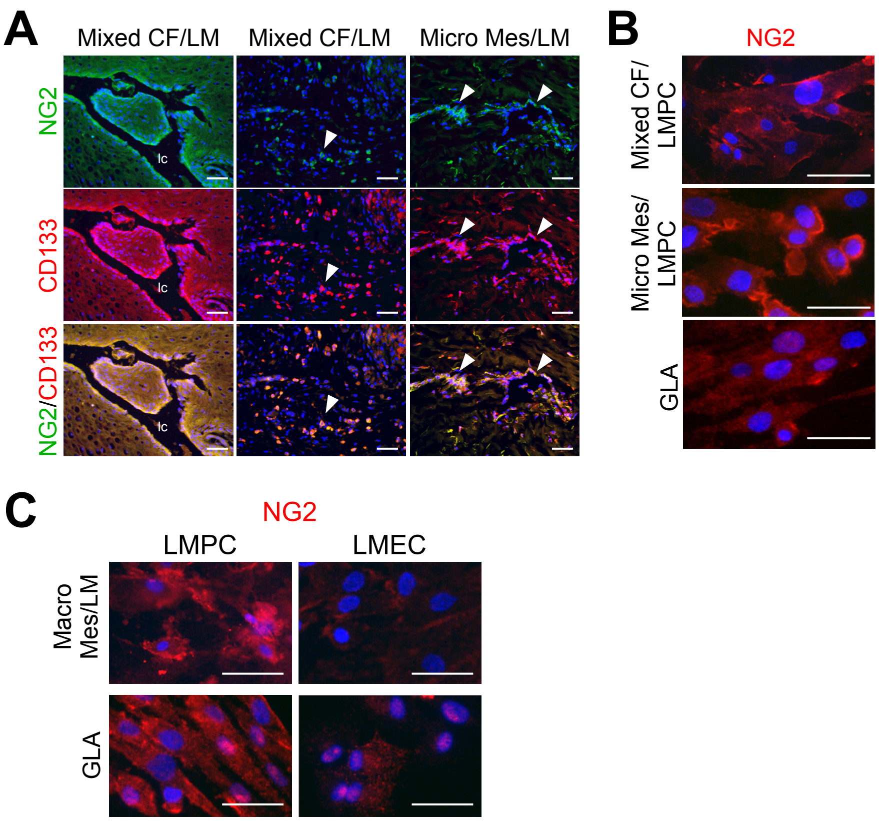

Figure S4. LMPCs expressed NG2. (**A**) NG2 and CD133 staining of mixed cervical facial (Mixed CF) and microcystic mesenteric (Micro Mes) LM tissues. White arrowheads mark abnormal lymphatic vessels. (**B**) NG2 staining of $CD133^+$ LM cells isolated from Mixed CF LM, Micro Mes LM tissues and generalized lymphatic anomaly (GLA) specimens. (**C**) NG2 staining of patient-matched $CD133^+$ LMPCs and $CD133^-$ LMECs isolated from macrocystic mesenteric (Macro Mes) LM and GLA. Scale bars: 50 μm. lymphatic channel (lc)

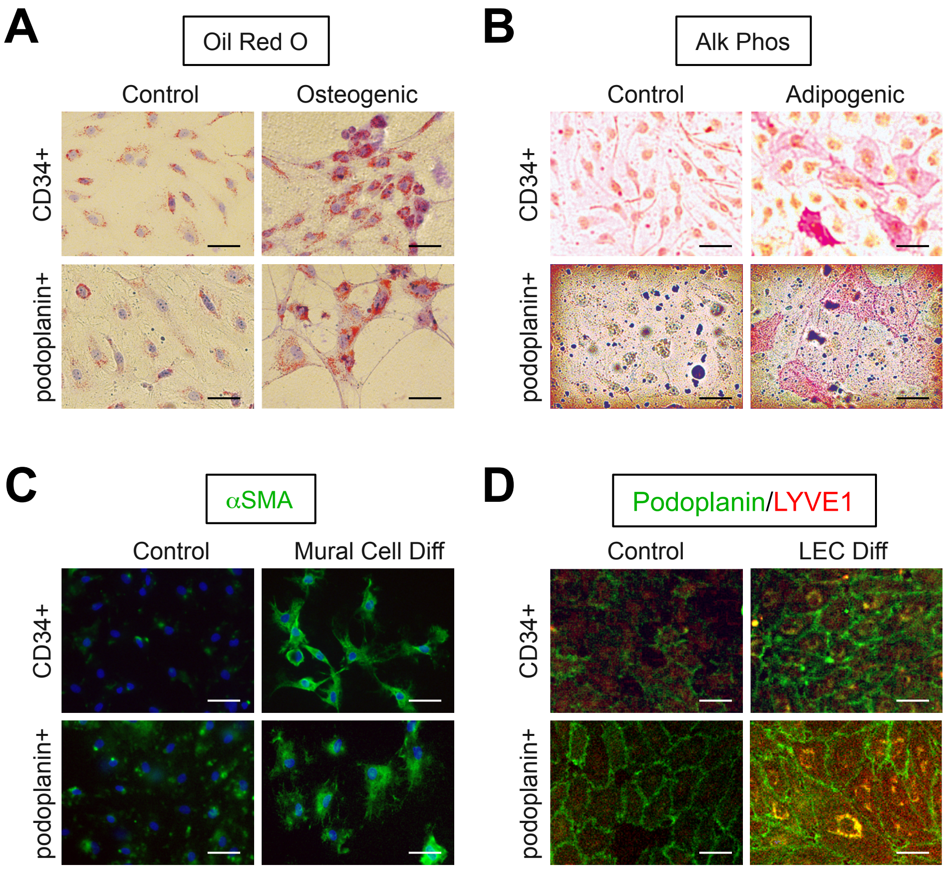

Figure S5. CD34+ and podoplanin+ LMPCs were multipotent. LMPCs isolated from a microcystic subcutaneous were sorted for CD34 or podoplanin positivity and induced to differentiate into fat, bone, VSMCs and LECs. (**A**) Oil Red O staining of $CD34^{+}$ or $podoplanin^{+}$ LMPCs after 2 weeks in growth media (control) or adipogenic media. (**B**) Alkaline phosphatase (Alk Phos) staining of $CD34^{+}$ or $podoplanin^{+}$ LMPCs after 2 weeks in growth media (control) or osteogenic media. (**C**) Alpha smooth muscle actin (αSMA) staining of $CD34^{+}$ or $podoplanin^{+}$ LMPCs after 2 weeks in growth media (control) or mural cell differentiation (Diff) media. (**D**) Podoplanin and LYVE1 staining of $CD34^{+}$ or $podoplanin^{+}$ LMPCs after 2 weeks in growth media (control) or LEC differentiation (Diff) media. Scale bars: 50 μm.

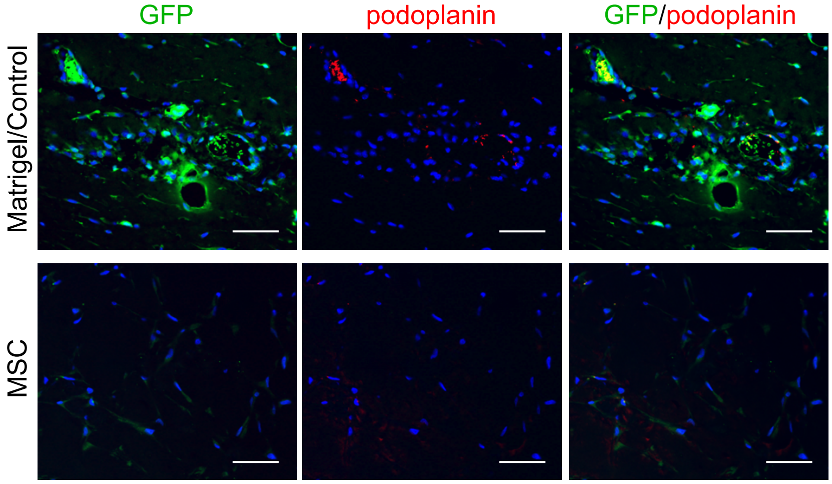

Figure S6. Analysis of control implants. Matrigel alone or MSCs suspended in Matrigel was implanted into GFP-expressing immunocompromised mice. GFP (host cell) and podoplanin staining of xenograft sections. Scale bars: 50 μm.
